# BTK Inhibitor Synergizes With CD19‐Targeted Chimeric Antigen Receptor‐T Cells in Patients With Relapsed or Refractory B‐Cell Lymphoma: An Open‐Label Pragmatic Clinical Trial

**DOI:** 10.1002/cam4.71321

**Published:** 2025-10-22

**Authors:** Wenjing Luo, Yinqiang Zhang, Chenggong Li, Jia Xu, Zhuolin Wu, Xindi Wang, Yun Kang, Danying Liao, Haiming Kou, Wei Xie, Wei Xiong, Jun Deng, Heng Mei, Yu Hu

**Affiliations:** ^1^ Institute of Hematology Union Hospital, Tongji Medical College, Huazhong University of Science and Technology Wuhan China; ^2^ Hubei Clinical Medical Center of Cell Therapy for Neoplastic Disease Wuhan China; ^3^ Key Laboratory of Biological Targeted Therapy (Huazhong University of Science and Technology), Ministry of Education Wuhan Hubei China; ^4^ Wuhan Sian Medical Technology Co., Ltd. Wuhan China

**Keywords:** B‐cell lymphoma, Bruton tyrosine kinase inhibitor, chimeric antigen receptor‐T cell, pragmatic clinical trial

## Abstract

**Background:**

CD19‐targeted chimeric antigen receptor‐T cell (CART19) therapy is clinically effective in patients with relapsed or refractory B‐cell lymphoma (BCL), but treatment failure and recurrence need to be overcome. Preclinical studies demonstrated that Bruton tyrosine kinase inhibitor (BTKi) improved the efficacy of CART19 therapy.

**Methods:**

We designed this open‐label, non‐randomized pragmatic clinical trial. The primary end point was safety, and the secondary end point was clinical response.

**Results:**

Thirty‐seven patients included were assigned to CART19 monotherapy (*n* = 24) or CART19 combined with BTKi (*n* = 13) group on their own accord. Grade 1–2 and grade 3 cytokine release syndrome occurred in 43.2% and 2.7% of patients, respectively. One patient experienced grade 3 neurotoxicity. The most common severe adverse events were hematological toxicities, including neutropenia (in 97.3% of patients), thrombocytopenia (in 40.5%), and anemia (in 43.2%). The adverse effects were comparable between the two groups. The best objective response rates were 84.6% vs. 66.7% (*p* > 0.05) in patients with and without BTKi, and the best complete response rates were 61.5% vs. 25.0% (*p* < 0.05). The combination of BTKi significantly prolonged the overall survival but did not affect the progression‐free survival or the duration of response. T cells of patients treated with BTKi were predisposed to early differentiation and less exhaustion 3 months after CART19 infusion. Single‐cell RNA sequencing analysis demonstrated that T cells were dysfunctional at relapse.

**Conclusion:**

BTKi combined with CART19 induced better outcomes with good safety profiles in patients with BCL.

**Trial Registration:**

ClinicalTrials.gov identifier: NCT05020392

## 1 Introduction

B‐cell lymphoma (BCL) represents the most common lymphoid malignancy [1]. In recent years, CD19‐targeted chimeric antigen receptor‐T cell (CART19) therapy has achieved breakthroughs, and four CART19 products have been approved by the US Food and Drug Administration (FDA) for patients with relapsed or refractory (R/R) BCL [2, 3, 4, 5]. Nevertheless, over 20% of patients still do not benefit from CART19 therapy, and over 60% of patients will experience post‐treatment progression or relapse [6, 7, 8, 9, 10].

Intrinsic chimeric antigen receptor‐T (CAR‐T) cell dysfunction and suppressive tumor microenvironment (TME) might be attributable reasons for CART19 therapy failure [11]. Strategies are needed to improve CART19 therapy, and combination treatment emerges as a potential solution. Checkpoint inhibitors [12], immunomodulatory agents [13], epigenetic drugs [14], and kinase inhibitors [15, 16] can synergize with CART19. Among them, Bruton tyrosine kinase inhibitor (BTKi) exhibited good safety and efficacy profiles in lymphoma, and ibrutinib (IB) was the first BTKi approved by the FDA for application in patients with mantle cell lymphoma (MCL) [17, 18, 19].

IB promoted T and CAR‐T cell proliferation, enriched less‐differentiated phenotype, and downregulated expression of exhaustion markers [16, 20, 21, 22]. The release of cytokines derived from CAR‐T cells was reduced by IB addition in vitro [16]. Additionally, IB could regulate cross‐talks in the TME through BTK‐dependent and ‐independent mechanisms [22, 23, 24]. Clinical trials supported that CART19 in combination with IB led to better responses and a lower grade of cytokine release syndrome (CRS) in patients with R/R chronic lymphocytic leukemia (CLL) and MCL [25, 26, 27, 28, 29]. Moreover, two novel BTKis, zanubrutinib (ZB) and orelabrutinib (OB), exert higher selectivity on BTK, and the combination of them with CART19 therapy needs further exploration [30, 31]. We previously reported that the phenotype and functionality of CART19 were modulated by the three BTKis in vitro and in mice models [24]. Here, we launched this open‐label pragmatic clinical trial (PCT) to prospectively investigate the synergism of BTKi and CART19 therapy in patients with BCL.

## 2 Materials and Methods

### 2.1 Clinical Trial Design and Participants

This open‐label, non‐randomized PCT was designed to evaluate the safety and efficacy of BTKi combined with CART19 in patients with BCL (NCT05020392). This study was approved by the Ethics Committee of the Union Hospital affiliated to Huazhong University of Science and Technology. All included patients provided written informed consents in accordance with the Declaration of Helsinki.

**FIGURE 1:**
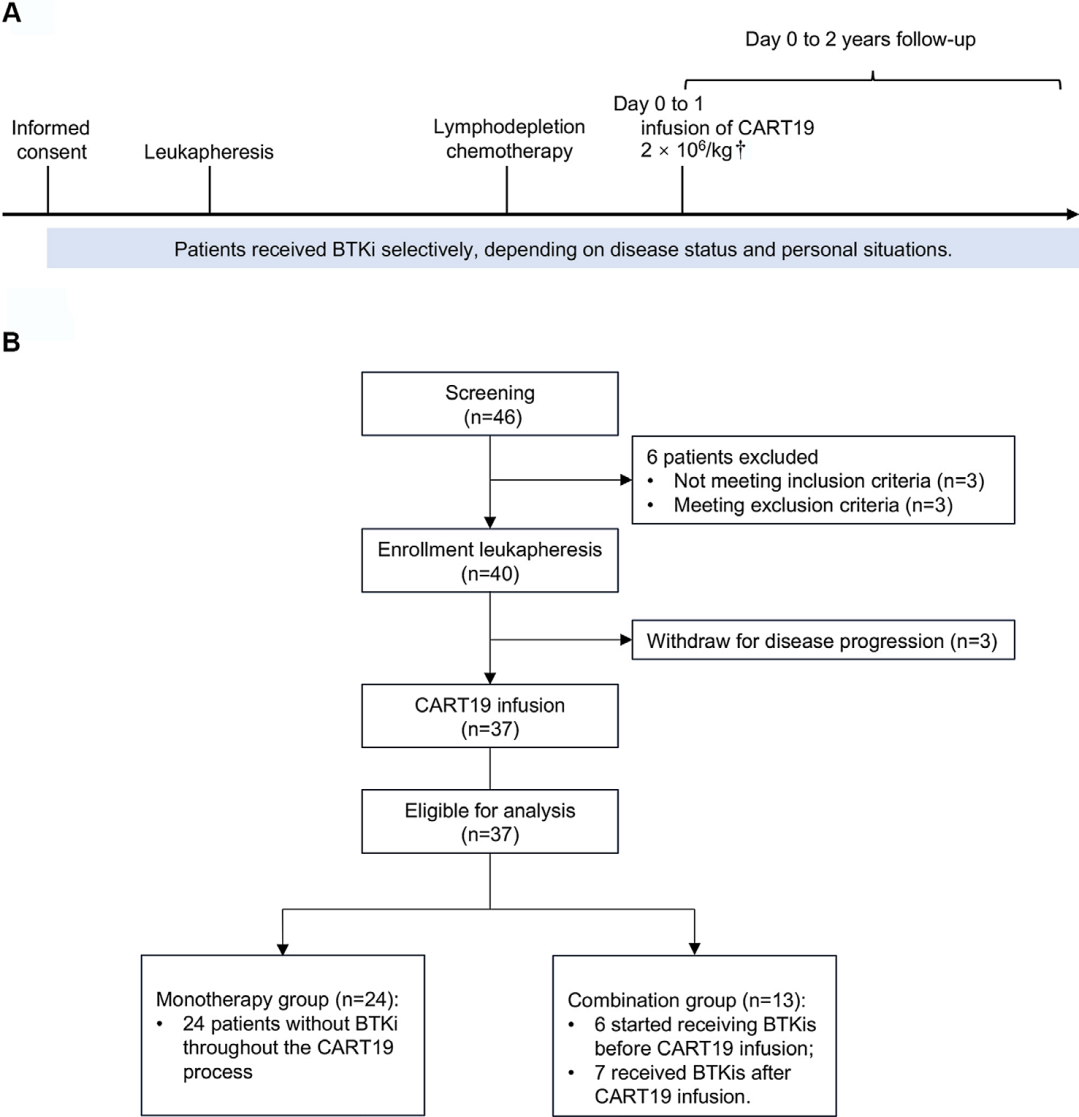
Study design and clinical trial profile. (A) Patients signed informed consent, underwent leukapheresis, lymphodepleting chemotherapy, and infusion of CART19. The included patients received BTKi after enrollment on their own accord. †Patient 10 received CART19 on day 0 and day 28, respectively. (B) The flow chart of the clinical trial.

Eligible patients must age ≥ 18 and ≤ 70 years and be diagnosed with R/R CD19‐positive BCL. Previous CAR‐T cell therapy was not allowed. After screening, patients received BTKi selectively, which depends on both disease status and personal situations, including patients' willingness, complications, and economic conditions (Figure 1A). Fludarabine‐based lymphodepletion chemotherapy was followed by CART19 infusion on day 0 to 1.

### 2.2 CART19 Manufacture

The CAR contained a humanized anti‐CD19 single‐chain variable fragment and a 4‐1BB costimulatory molecule (Figure S1). The detailed information and manufacturing process of CD19 CAR‐T cells was previously described [32]. Briefly, T cells from apheresis products of patients were isolated and transduced with lentivirus encoding anti‐CD19 CAR and expanded in vitro. The standard of release tests was provided in Supplementary methods.

### 2.3 Outcomes Measurement

The primary end point was the safety of CART19 in combination with or without BTKi. Adverse effects (AEs) were recorded and assessed according to the National Institutes of Health Common Terminology Criteria for Adverse Events Version 5.0 (http://ctep.cancer.gov/). Specifically, CRS and neurotoxicity were graded by the criteria of the American Society for Transplantation and Cellular Therapy [33]. The secondary end points were overall response rate (ORR), overall survival (OS), progression‐free survival (PFS), and duration of response (DOR). Responses were evaluated per Lugano criteria [34]. Other objectives included the pharmacokinetics of CAR‐T cells and the phenotype of T cells. Expansion and persistence of CART19 in peripheral blood (PB) were monitored by flow cytometry (FCM) and droplet digital polymerase chain reaction (ddPCR). T cell exhaustion and differentiation were assessed by multiparameter FCM [35]. The antibodies and primers used were provided in Tables S1 and S2.

### 2.4 Single‐Cell RNA Sequencing and Data Processing

Peripheral blood mononuclear cells (PBMCs) were isolated by Ficoll density gradient sedimentation. Single‐cell RNA sequencing (scRNA‐seq) libraries of PBMCs were prepared using SeekOne Digital Droplet Single Cell 3′ library preparation kit according to the manufacturer's protocol. The indexed sequencing libraries were sequenced on Illumina NovaSeq6000 with PE150 read length at Seekgene (Beijing, China).

We used Seeksoul Tools Online to process sequence data and aligned reads to human GRCh38. Downstream analyses were processed by the Seurat package v4.3.0.1 in R software v4.3.1. Integrative analysis was performed using the Harmony package v3.17. The data were scaled and dimensionally reduced with principal components analysis and t‐distributed stochastic neighbor embedding (t‐SNE). Significant differentially expressed genes (DEGs) were identified as log2 fold‐change > 0.25 and adjusted *p* < 0.05. Enrichment analyses of DEGs were implemented by the clusterProfiler package v4.8.2. The Monocle package v2.28.0 was used to determine the potential lineage differentiation.

### 2.5 Statistical Analysis

Descriptive statistics included means with a 95% confidence interval and medians with a range. Comparisons of continuous variables were made using a two‐tailed t test. Changes in cytokines and CART19 kinetics in two groups were analyzed by a two‐way ANOVA. Differences in categorical variables were performed by the Chi‐square statistic or Fisher's exact test as appropriate. OS, PFS, and DOR probabilities were calculated by the Kaplan–Meier method and were compared using the log‐rank test. All *p* values were two‐sided, and the results were considered statistically significant when *p* < 0.05. Plots and statistical analysis were performed with GraphPad Prism 9 (San Diego, California, USA) or R 4.0.3 (www.r‐project.org).

## 3 Results

### 3.1 Characteristics of Patients

Until February 29, 2024, of the 46 patients screened, 37 were treated and assigned to the CART19 monotherapy (*n* = 24) or CART19 combined with BTKi (*n* = 13) group (Figure 1B). Baseline demographics, disease characteristics, and the usage of BTKi were listed in Table 1 and Table S3, respectively. In our study, 27 (73.0%) patients were diagnosed with diffuse large B‐cell lymphoma (DLBCL), 4 (10.8%) were follicular lymphoma (FL), 4 (10.8%) were transformed FL, 1 (2.7%) was Burkitt lymphoma, and 1 (2.7%) was MCL. Dual expressor lymphoma was observed in 11 (29.7%) patients. Thirty‐one (83.8%) had stage III or IV disease according to Ann Arbor staging, and 27 (73.0%) had extranodal infiltration. The median prior lines of treatment were 3 (range, 2–9), and 25 (67.6%) patients were primary refractory. Baseline characteristics were comparable between the two groups.

**TABLE 1:** Comparison of baseline characteristics between the two groups.

Characteristics	All (*n* = 37)	Monotherapy (*n* = 24)	Combination (*n* = 13)	*p*
Median age (range)	57 (24–69)	58.5 (30–69)	53 (24–63)	0.3115
Male, *n* (%)	19 (51.4)	13 (54.2)	6 (46.2)	0.7374
DLBCL, *n* (%)	27 (73.0)	17 (70.8)	10 (76.9)	> 0.9999
GCB type^a^, *n* (%)				0.3998
Yes	8 (29.6)	6 (35.3)	2 (20.0)	
No	15 (55.6)	8 (47.1)	7 (70.0)	
Double expressor lymphoma^b^, *n* (%)				> 0.9999
Yes	11 (29.7)	7 (29.2)	4 (30.8)	
No	19 (51.4)	12 (50.0)	7 (53.8)	
Ann Arbor stage (III–IV), *n* (%)	31 (83.8)	20 (83.3)	11 (84.6)	> 0.9999
Extranodal organ involvement, *n* (%)	27 (73.0)	19 (79.2)	8 (61.5)	0.4645
BM involvement, *n* (%)	5 (13.5)	4 (16.7)	1 (7.7)	0.6378
Bulky ≥ 5 cm, *n* (%)	16 (43.2)	11 (45.8)	5 (38.5)	0.7386
ECOG, *n* (%)				> 0.9999
0–1	30 (81.1)	19 (79.2)	11 (84.6)	
2	7 (18.9)	5 (20.8)	2 (15.4)	
Prior lines ≥ 3, *n* (%)	22 (59.5)	15 (62.5)	7 (53.8)	0.5078
Primary refractory, *n* (%)	25 (67.6)	16 (66.7)	9 (69.2)	> 0.9999
Prior auto‐HSCT, *n* (%)	5 (13.5)	3 (12.5)	2 (15.4)	> 0.9999
Median LDH (U/L; range)	215 (25–1890)	215.5 (25–1890)	215 (91–1437)	0.7607

*Note:* Not available data: cell‐of‐origin of DLBCL (*n* = 6); double expression (*n* = 7). LDH normally ranges from 120 to 246 U/L.

Abbreviations: BM, bone marrow; DLBCL, diffuse large B‐cell lymphoma; ECOG, Eastern Cooperative Oncology Group; GCB, germinal center B‐cell‐like; HSCT, hematopoietic stem cell transplantation; LDH, lactate dehydrogenase.

^a^ The percentage of GCB type was calculated in patients with DLBCL.

^b^ Lymphoma with overexpression of MYC and BCL2 proteins by immunohistochemistry, regardless of detectable translocation by fluorescence in situ hybridization.

### 3.2 Safety

**FIGURE 2:**
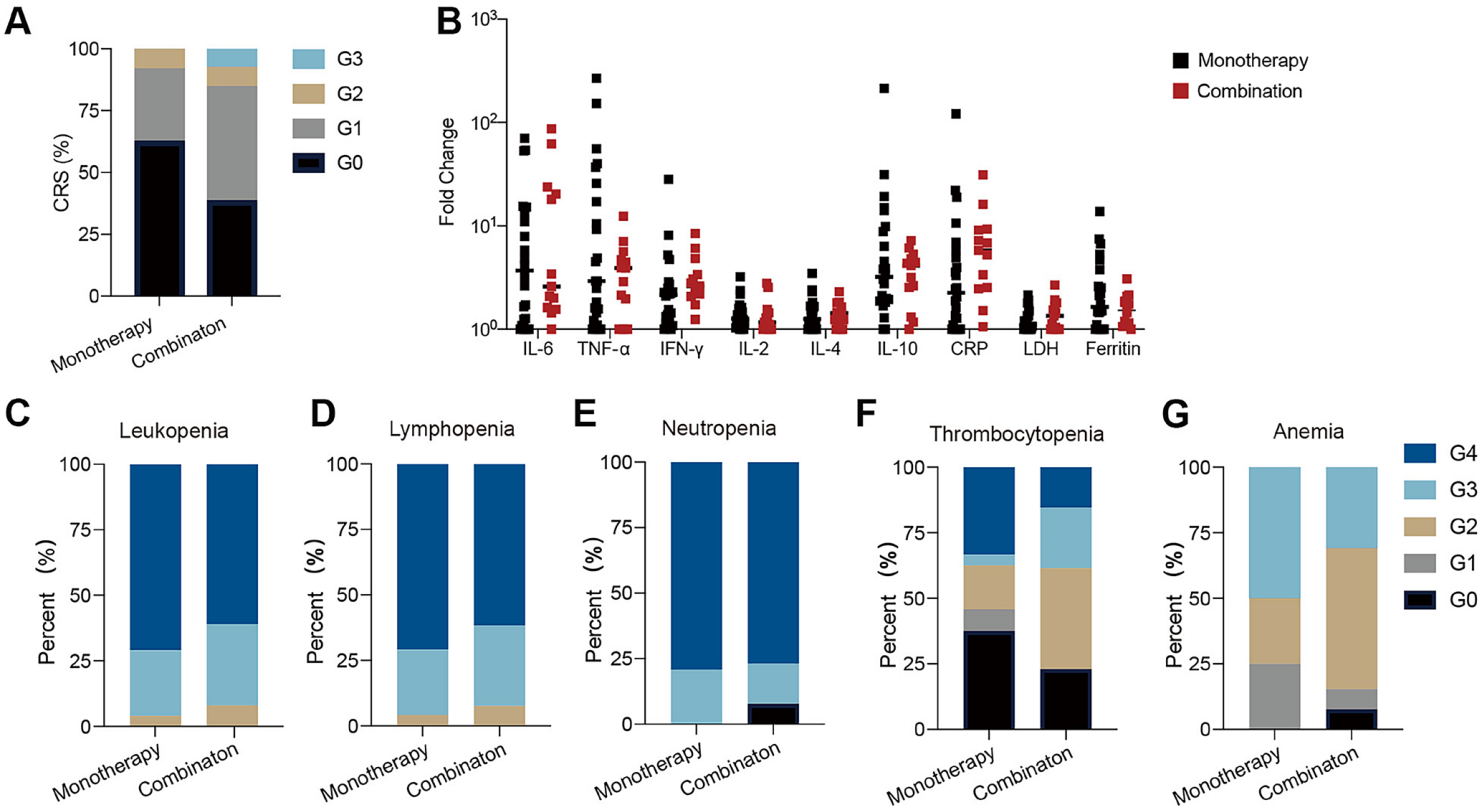
Adverse effects after CART19 infusion. (A) Comparison of CRS grade between the monotherapy and combination groups. (B) Fold‐change of inflammatory factors from baseline to peak. (C–G) Comparisons of leukopenia (C), lymphopenia (D), neutropenia (E), thrombocytopenia (F), and anemia (G) grade between the two groups. CRP, C‐reaction protein; G, grade; IFN, interferon; IL, interleukin; LDH, lactate dehydrogenase; TNF, tumor necrosis factor.

CRS occurred in 17 patients (45.9%), of whom patient 32 suffered from grade 3. No statistical significance was noted between monotherapy and combination groups (37.5% and 61.5%; Figure 2A). Twelve patients received glucocorticoids, and one received tocilizumab for the management of CRS. Patient 28 experienced grade 3 neurotoxicity, which was manageable with glucocorticoids and supportive treatments. The dynamics of inflammatory markers (interleukin (IL)‐6, tumor necrosis factor‐α, interferon‐γ, IL‐2, IL‐4, IL‐10, C‐reactive protein, lactate dehydrogenase, and ferritin) post‐infusion and their fold changes from baseline to peak did not show statistical significance between the two groups (Figure 2B and Figure S2).

The hematological toxicities were the most common AEs. Grade ≥ 3 leukopenia, lymphopenia, neutropenia, thrombocytopenia, and anemia were 91.9% (34/37), 94.6% (35/37), 97.3% (36/37), 40.5% (15/37), and 43.2% (16/37), respectively. The median time for patients with grade 3–4 cytopenia that recovered to grade 0–2 was 14 days for leukopenia, 13 days for lymphopenia, 14 days for neutropenia, 60 days for thrombocytopenia, and 45 days for anemia (Figure S3A). The occurrence rates of grade 3–4 leukopenia, lymphopenia, neutropenia, thrombocytopenia, and anemia were 91.7% (22/24) vs. 92.3% (12/13), 95.8% (23/24) vs. 92.3% (12/13), 100.0% (24/24) vs. 92.3% (12/13), 37.5% (9/24) vs. 46.2% (6/13), and 50.0% (12/24) vs. 30.8% (4/13) in monotherapy and combination groups, respectively (Figure 2C–G). There were no differences between the two groups in the incidences or duration of grade 3–4 cytopenias (Figure S3B). A grade 2 gastrointestinal bleeding event occurred once in patient 11, manifested as melena. The patient had a lesion in the abdominal cavity and recovered after the intravenous steroid and symptomatic treatment. Hypo‐immunoglobulin gammaglobulinemia (IgG) and B‐cell aplasia were common in all patients throughout the whole therapy (Figure S4A,B). In the most recent available follow‐up, IgG of patient 25 in the monotherapy group recovered to normal level; B‐cell recovery, defined as B cells ≥ 3% of lymphocytes in PB [36], was observed in two patients in each group.

In monotherapy group, six patients (25.0%) experienced seven infections within 1 month, including one viral infection of grade 2, three bacterial infections of grade 3, and three fungal infections of grade 3. In combination group, two patients (15.0%) suffered from two bacterial infections of grade 3 within 1 month. Infections were manageable and no statistical significance was observed between the two groups.

### 3.3 Efficacy

**FIGURE 3:**
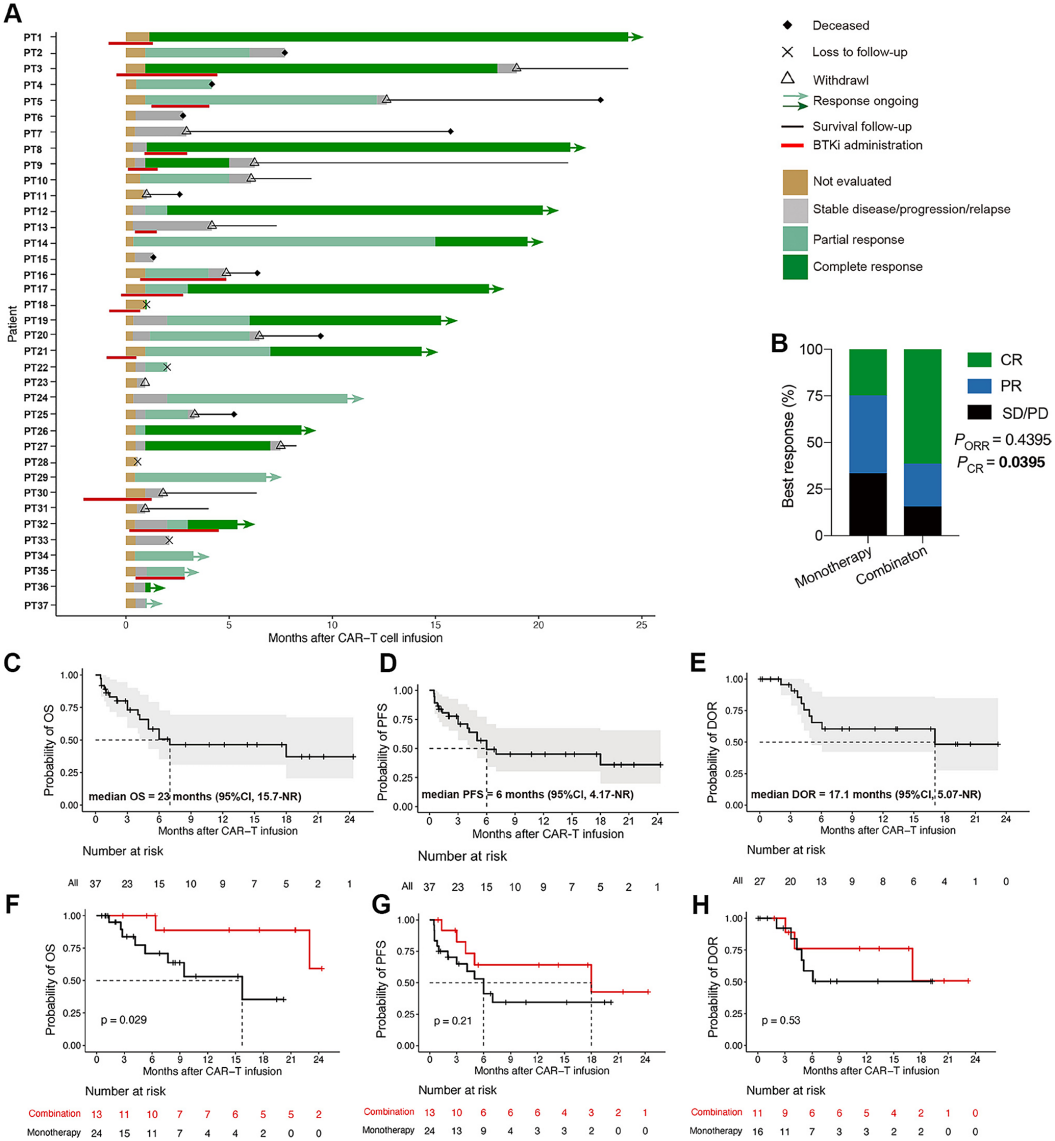
CART19 with concurrent BTKis induced better outcomes in patients with BCL. (A) Swimmer's plot of the 37 treated patients. The red bars under the swim lane denoted the timelines of BTKi administration. Patients 17, 18, and 21 received ibrutinib, patients 1, 3, 9, 13, 30, and 35 received zanubrutinib, and patients 5, 8, 16, and 32 received orelabrutinib. (B) The best response between the monotherapy and combination groups. (C–E) Kaplan–Meier curves of the OS (C), PFS (D) and DOR (E) of all treated patients. (F–H) Kaplan–Meier curves and HR estimates of the OS (F), PFS (G) and DOR (H) between the two groups. CR, complete response; DOR, duration of response; NR, not reached; OS, overall survival; PD, progression disease; PFS, progression free survival; PR, partial response; PT, patient; SD, stable disease.

Of 37 patients, 14 (37.8%) achieved complete response (CR), 13 (35.1%) obtained partial response, and 10 (27.0%) did not respond (Figure 3A). The best ORRs were 84.6% and 66.7% (*p* = 0.4395) in combination and monotherapy groups, and the best CR rates were 61.5% and 25.0% (*p* = 0.0395), respectively (Figure 3B). The median duration of BTKi administration was 2.4 months (range, 1.1–4.9 months) and the detailed timelines were depicted in Figure 3A with red bars.

At a median follow‐up of 6.8 (range, 0.6–24.3) months, the median OS, PFS, and DOR were 23, 6, and 17.1 months respectively (Figure 3C–E). The combination of BTKi significantly prolonged OS (*p* = 0.029) but did not affect PFS (*p* = 0.21) or DOR (*p* = 0.53; Figure 3F–H), which was also observed in patients with DLBCL (Table 2). Furthermore, we explored the different effects of IB, ZB, and OB on OS, PFS, and DOR; however, no statistical significance was noted (Figure S5). Additionally, there were no differences in OS, PFS, or DOR between patients who received BTKi before (*n* = 6) and after (*n* = 7) CART19 infusion (Figure S6).

**TABLE 2:** Survival analysis of different subgroups.

Subgroups		OS (months)			PFS (months)			DOR (months)		
		Median (95% CI)	HR (95% CI)^a^	*p*	Median (95% CI)	HR (95% CI)^a^	*p*	Median (95% CI)	HR (95% CI)^a^	*p*
All	Monotherapy	15.7 (7.7–NR)	0.13 (0.02–1.10)	**0.029**	6 (3–NR)	0.52 (0.18–1.46)	0.21	NR (4.83–NR)	0.64 (0.16–2.60)	0.53
	Combination	NR (23–NR)			18 (5–NR)			NR (17.07–NR)		
DLBCL	Monotherapy	15.7 (5.23–NR)	0.16 (0.02–1.29)	**0.049**	6 (3–NR)	0.69 (0.23–2.06)	0.51	NR (5.07–NR)	0.81 (0.18–3.63)	0.78
	Combination	NR			18 (4–NR)			17.1 (4.07–NR)		
Dual expressor lymphoma	Monotherapy	7.7 (5.23–NR)	0.31 (0.03–3.48)	0.32	6 (3–NR)	0.59 (0.09–3.65)	0.56	5.07 (2.07–NR)	0.76 (0.10–5.51)	0.79
	Combination	NR (6.37–NR)			5 (4–NR)			4.07 (3.07–NR)		
Ann Arbor stage III–IV	Monotherapy	NR (5.23–NR)	0.24 (0.03–2.05)	0.16	7 (4.17–NR)	0.59 (0.18–1.92)	0.37	NR (5.07–NR)	0.49 (0.09–2.58)	0.39
	Combination	NR (23–NR)			18 (4–NR)			NR (17.07–NR)		
With extranodal involvement	Monotherapy	9.43 (5.23–NR)	0.21 (0.03–1.76)	0.12	5 (2–NR)	0.70 (0.23–2.20)	0.54	6.07 (4.3–NR)	1.21 (0.29–4.95)	0.79
	Combination	NR (6.37–NR)			18 (4–NR)			17.07 (4.07–NR)		
Prior lines ≥ 3	Monotherapy	15.7 (4.17–NR)	0.17 (0.02–1.48)	0.074	4.17 (1–NR)	0.29 (0.06–1.32)	0.085	6.07 (3.67–NR)	0.37 (0.04–3.33)	0.36
	Combination	23 (23–NR)			18 (4–NR)			17.07 (17.07–NR)		
Primary refractory	Monotherapy	15.7 (5.23–NR)	0.21 (0.02–1.73)	0.11	3 (0.83–NR)	0.40 (0.11–1.44)	0.15	NR (5.07–NR)	0.79 (0.13–4.80)	0.80
	Combination	23 (23–NR)			18 (4–NR)			17.1 (NR–NR)		

*Note:* Some median survivals were not reached (NR) due to the short follow‐up and small sample size. The *p* value was calculated using the log‐rank test, and the bold *p*‐values indicated statistical significance (*p* < 0.05).

Abbreviations: DLBCL, diffuse large B‐cell lymphoma; DOR, duration of response; HR, hazard ratio; OS, overall survival; PFS, progression‐free survival.

^a^ Patients who did not receive BTKis were considered as the reference group.

### 3.4 Pharmacokinetics of CART19 and Multiparametric Flow Cytometric Analysis

**FIGURE 4:**
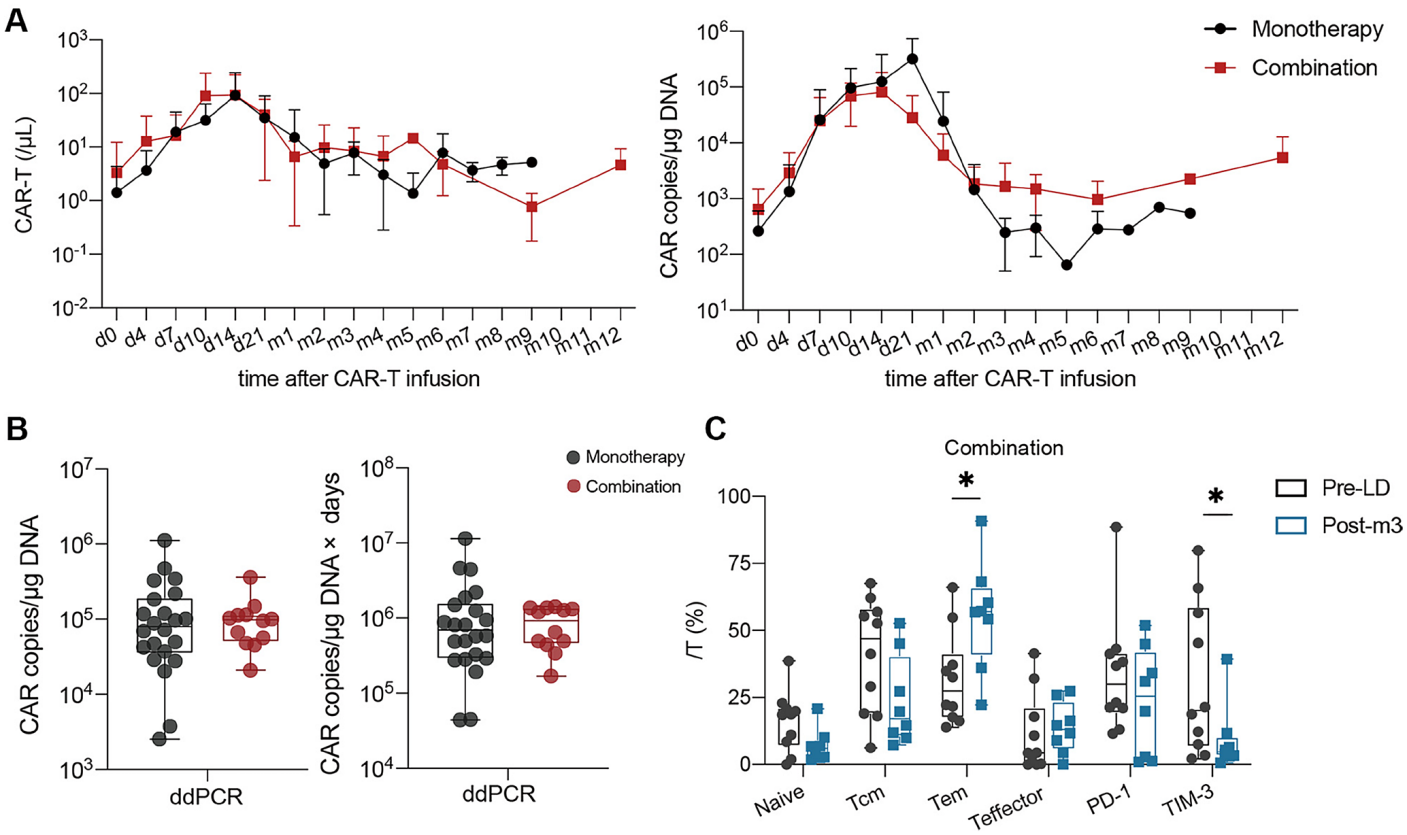
Kinetics of CART19 and multiparametric flow cytometric analysis. (A) Pharmacokinetics of CART19 in PB detected by flow cytometry (left), and kinetics of CAR copies in PB detected by ddPCR (right). (B) Comparisons of peak CAR copies/μg DNA and the area under the expansion curve within the first 28 days between the two groups. (C) The T cell differentiation and exhaustion at the two timepoints in the combination group. d, day; m, month; ddPCR, droplet digital polymerase chain reaction; Tcm, central memory T; Tem, effector memory T; LD, lymphodepletion. *p < 0.05.

The median transduction efficiency of the CART19 products was 44.5% (range, 17.1%–79.7%) and the median ratio of CD4^+^ to CD8^+^ CART19 was 1.58 (range, 0.24–8.61). The characteristics of infused CAR‐T cells were comparable between the two groups (Figure S7). Quantitation of CART19 was performed by FCM and ddPCR. CART19 (*n* = 35) peaked at a median of 50,250 (range, 2440–521,930) cells/mL by FCM on day 14; CAR copies (*n* = 34) peaked at a median of 91,500 (range, 2550–1,119,500) copies/μg DNA by ddPCR on day 10 (Figure 4A). Peak expansion and the area under the expansion curve in the first 28 days after infusion were not significantly associated with the combination of BTKi (Figure 4B and Figure S8). Immunophenotypic analysis of circulating T cells pre‐ and post‐infusion revealed that the effector memory T cell (Tem) subsets increased and TIM3^+^ T cells decreased 3 months after CART19 infusion in the combination group (Figure 4C), but not in the monotherapy group (Figure S9).

### 3.5 Single‐Cell RNA Sequencing

**FIGURE 5:**
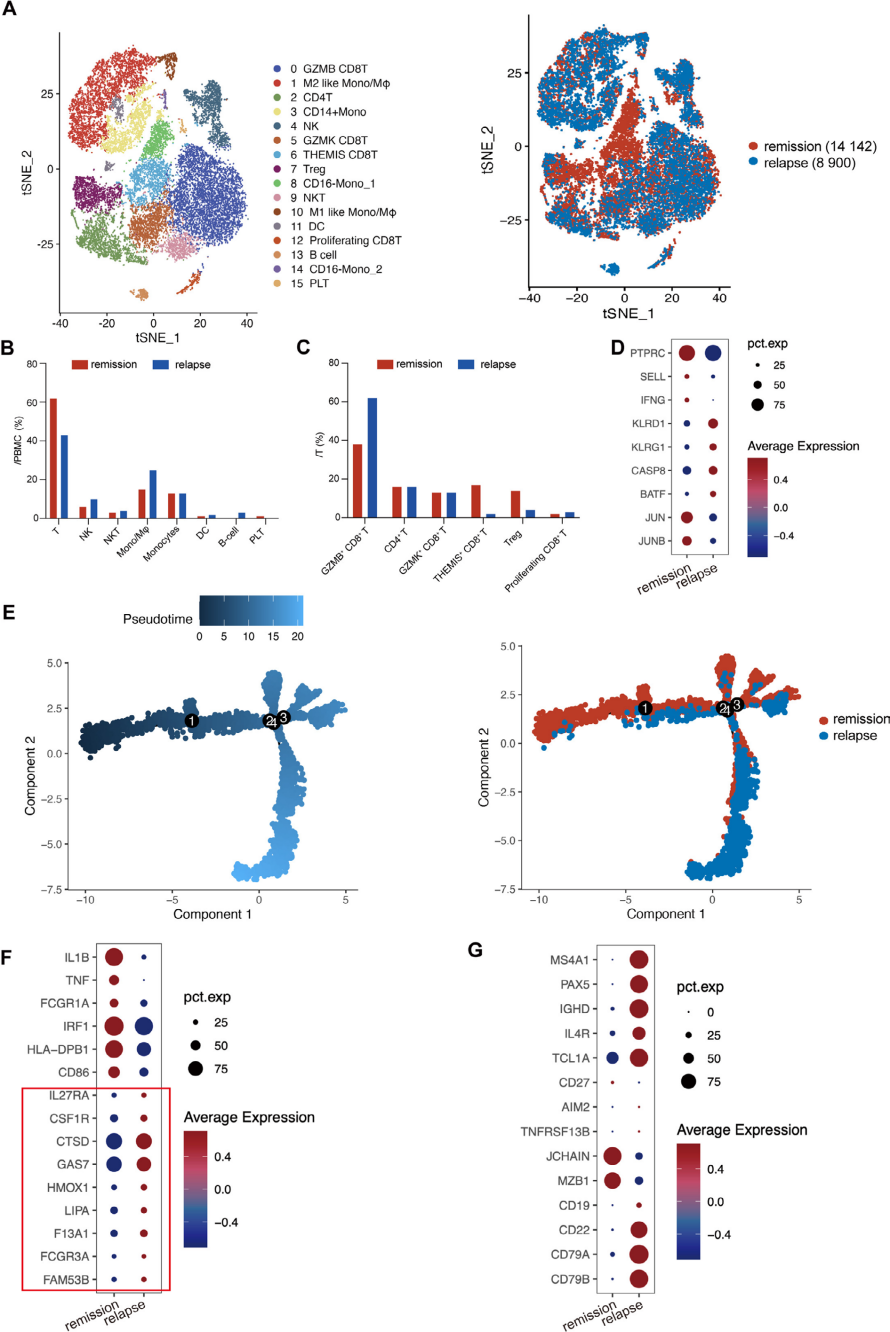
Single‐cell RNA sequencing analysis of patient 3 at remission and relapse. (A) t‐SNE plots with shared nearest neighbor clustering of 14,142 and 8900 peripheral blood mononuclear cells (PBMCs) at remission and relapse respectively, colored by 16 transcriptional clusters (left). (B) Proportion of various cell populations in PBMCs at remission and relapse. (C) Proportion of T‐cell subsets at remission and relapse. (D) Dot plot comparing the relative expression of nine genes in T cells at remission and relapse. (E) Pseudotime analysis of two samples. (F) Dot plot comparing the relative expression of 15 genes in monocytes/macrophages at remission and relapse. Genes in the red box were associated with M2‐like macrophages. (G) Dot plot comparing the relative expression of 14 genes in B cells at remission and relapse. DC, dendritic cell; Mono, monocytes; Mφ, macrophages; NK, natural killer cell; NKT, natural killer T cell; PLT, platelet.

Patient 3 achieved CR at 1 month and kept durable remission until 18 months after infusion. To compare the immune status at remission and relapse, we performed scRNA‐seq analysis of PBMCs. Following quality control, unsupervised graph‐based clustering of 23,042 cells integrated from 2 samples identified 16 clusters (Figure 5A and Figure S10A). Compared with the sample at relapse, the sample at remission contained a higher percentage of T cells (61.99% vs. 42.72%) and a lower proportion of monocytes/macrophages (14.8% vs. 24.78%) and B cells (0.17% vs. 2.62%; Figure 5B).

T cells were divided into six clusters by t‐SNE analysis (Figure 5C and Figure S10B). We compared the transcriptional profiles of two specimens and observed that the sample at relapse downregulated genes associated with T cell memory subsets (*JUN* and *JUNB*) and upregulated dysfunctional markers (*CASP8* and *BATF*; Figure 5D). Additionally, pseudotime analysis revealed that CD8^+^ T cells at relapse were at a later differential stage compared with that at remission (Figure 5E). The percentage of GZMB^+^ CD8 T^+^ cluster 0 increased from 37.5% to 62.4% over time, while GZMK^+^ CD8^+^ T cluster 5 was unchanged between the two timepoints. In comparison with cluster 5, we found that cluster 0 expressed lower levels of genes related to T cell memory phenotype and proliferation and higher levels of genes associated with apoptosis and exhaustion (Figure S11), consistent with a previous study [37]. *THEMIS*, a critical gene involved in T cell receptor (TCR) signal transduction, was associated with T cell proliferation and inflammatory potential [38, 39]. The THEMIS^+^ CD8 T^+^ cluster 6 was largely lost at relapse (Figure 5C). DEGs between cluster 6 and the other T cell clusters were enriched in the TCR signaling pathway (Figure S12).

Monocytes/macrophages (cluster 1 and 10) at relapse highly expressed M2‐related genes (Figure 5F), demonstrating the immune disorders. B‐cell recovery indicated the lack of functional CAR‐T cells [25]. B cells at remission accounted for 0.2% and exhibited markedly higher levels of genes associated with plasma cells, including *JCHAIN* and *MZB1* (Figure 5G). B cells at relapse accounted for 2.6% and were characterized by robust expression of genes *IL4R* and *TCL1A* associated with naïve phenotypes. Notably, the percentage of B cells expressing *CD19* was lower than that expressing *CD22*, *CD79A*, or *CD79B* at relapse.

## 4 Discussion

CART19 therapy has shown remarkable clinical efficacy in patients with R/R BCL, leading to the first FDA approval for gene‐editing therapies and boosting the development of cancer immunotherapy. Preclinical and clinical studies demonstrated that BTKi could enhance the efficacy of CART19 therapy in patients with R/R CLL and MCL [25, 26, 27]. However, prospective clinical trials are lacking to clarify the synergism of BTKi and CART19 in BCL.

Several studies revealed that BTKi could modulate the immunosuppressive TME in lymphoma, and patients administered continuous BTKi would benefit more from CART19 therapy [20, 22, 24, 27]. Therefore, patients with BTKi treatment at enrollment were admitted. Because relapse has remained a big obstacle for CAR‐T cell therapy, BTKi as maintenance treatment was also adopted. Our preclinical results demonstrated that IB, ZB, and OB all improved the effectiveness of CART19 in BCL [24]. Thus, the three BTKis were all acceptable, and patients could receive any one of them on their own accord, which depends on both disease status and personal reasons, including patients' willingness, complications, and economic factors. For instance, given the documented efficacy of BTKis in MYD88 mutant disease [40] and the favorable safety profile of OB [41], patient 32 with nasopharyngeal and maxillary sinus lesions harboring the MYD88 mutation received OB on day 5 after CART19 infusion. However, financial constraints necessitated discontinuation of oral OB 4.5 months after CART19 therapy.

Due to the difficulty of randomization, we conducted the non‐randomized interventional PCT, aiming to provide relatively high‐quality evidence from the real world [42, 43]. Nevertheless, the patient characteristics were comparable. CART19 therapy achieved the best ORRs of 44%–83% and the CR rates of 28%–65% in patients with R/R BCL [7, 8, 9, 10]. Patients in our study achieved comparable efficacy outcomes, with the best ORR and CR rates of 73.0% and 37.8%, respectively. BTKi combined with CART19 mediated a higher response. Moreover, we observed that BTKi prolonged the OS of patients. Recent research demonstrated that patients receiving BTKi before CART19 infusion obtained better clinical outcomes [44], which was not noted in our study. The different timing and duration of BTKi treatment across CART19 therapy might explain the distinct conclusions. Notably, three patients with concurrent IB all achieved CR, and two of them kept ongoing CR during follow‐up. Combining our previous findings that IB stood out in synergizing with CART19 [24], we speculated that the combination of CART19 therapy and IB would induce a better outcome for patients with BCL. Possibly due to the small sample size, IB, ZB, and OB did not show statistical significance on prognosis in these patients.

The hematological toxicities and CRS were the most common AEs but manageable, which was consistent with other studies [25, 26, 27, 28]. Patients exposed to BTKi suffered from hematological AEs, and IB administration might cause atrial fibrillation [17, 18, 45]. Fortunately, we observed that the addition of BTKi to CART19 therapy did not aggravate cytopenias or induce atrial fibrillation in patients with BCL. Previous articles revealed that the combination of BTKi with CART19 contributed to lower CRS severity and lower serum concentrations of CRS‐related cytokines [26, 29]. Meanwhile, it was reported that CRS grades were significantly greater in patients receiving the sequential use of IB and CAR‐T cells than those treated with CART19 alone [28], which might be attributed to IB‐enhanced CAR‐T cell functionality and subsequently promoting cytokine release. However, our results showed that CRS occurred comparably between the monotherapy and combination groups in patients with BCL. These discrepancies may reflect differences in CAR‐T cell products, disease type, tumor burden, and different BTKi administrations, which were related to CRS incidence and severity [24, 46].

Patient 3 obtained CR at 1 month but progressed at 18 months after infusion. scRNA‐seq analysis indicated that T cells at relapse obtained a more differentiated, exhausted, and dysfunctional immunotype. Moreover, monocytes/macrophages at relapse highly expressed M2‐related genes. Multiparameter FCM results indicated that BTKi decreased TIM3 expression on T cells and enriched Tem. Our previous study revealed that BTKi could prevent T cells from exhaustion and terminal differentiation and polarize macrophages into the M1 subtype [24]. Mechanistically, BTKis diminished IL‐2 inducible T cell kinase and CD3‐ζ phosphorylation of TCR and CAR, as well as downregulated the T cell activation‐associated JAK–STAT signaling pathway [24], thereby protecting CAR‐T cells from excessive activation and preserving their functionality. Meanwhile, BTKis‐mediated M2 macrophage reduction in a target‐dependent approach [24, 47, 48] would further augment CAR‐T cell activity [49]. The failure of ZB contributing to immunomodulation in patient 3 might be attributed to the patient stopping oral ZB at 4.4 months after CART19 infusion, and thus we supposed that long‐term maintenance of BTKi would benefit more to keep durable remission.

To our knowledge, this is the first PCT for prospectively evaluating the synergism of BTKi and CART19 in patients with BCL. However, the statistical power of our study may be constrained by the relatively small sample size. Furthermore, the non‐randomized design carries inherent limitations [50]. First, the lack of randomization resulted in the unbalanced sample size between the two groups. Second, unmeasured factors, particularly the socioeconomic status and treatment adherence, would impact both the BTKi administration and clinical outcomes, thereby introducing selection bias into the analysis. These limitations underscore the necessity for large, prospective, randomized clinical trials to validate the observed synergistic effects and establish optimal combination protocols.

## 5 Conclusion

Our study demonstrated that the combination of BTKi and CART19 induced superior efficacy in terms of response depth and long‐term survival. More early‐differentiated and less TIM3^+^ exhausted T cells were observed in patients receiving BTKi during CART19 therapy. Prolonging the duration of BTKi maintenance might contribute to the outcomes of patients with BCL through improving immune function.

## Author Contributions

**Wenjing Luo:** investigation, writing – original draft, visualization, methodology, validation, software, formal analysis, data curation. **Yinqiang Zhang:** visualization, validation, methodology, software, writing – review and editing. **Chenggong Li:** investigation, writing – review and editing. **Jia Xu:** investigation. **Zhuolin Wu:** investigation. **Xindi Wang:** investigation. **Yun Kang:** investigation. **Danying Liao:** supervision, resources. **Haiming Kou:** supervision, resources. **Wei Xie:** supervision, resources. **Wei Xiong:** resources. **Jun Deng:** supervision. **Heng Mei:** conceptualization, supervision, resources, funding acquisition, writing – review and editing, project administration. **Yu Hu:** conceptualization, supervision, resources, project administration.

## Ethics Statement

This study was approved by the Ethics Committee of the Union Hospital affiliated with Huazhong University of Science and Technology (no. [2021] 0711‐01).

## Consent

Informed consent was obtained from all patients before enrollment.

## Conflicts of Interest

Wei Xiong is employed in Wuhan Sian Medical Technology Co. Ltd., and the other authors declare no competing interests.

## Supporting information


**Data S1:** cam471321‐sup‐0001‐Supinfo.zip.

## Data Availability

The original data supporting the findings of this study are included in the article or Supporting Information.
