# Supplementary material for: BTK Inhibitor Synergizes With CD19‐Targeted Chimeric Antigen Receptor‐T Cells in Patients With Relapsed or Refractory B‐Cell Lymphoma: An Open‐Label Pragmatic Clinical Trial
Source: Cancer Med. 2025 Oct 22;14(20):e71321. doi: 10.1002/cam4.71321 (PMC12541673; doi:10.1002/cam4.71321)

**Supplementary figures S1-S12**

**Figure S1. The structure of anti-CD19 CAR.** LTR, long terminal repeats; EF, elongation factor; GM-CSF, granulocyte macrophage-colony stimulating factor; scFv, single-chain variable fragment; TM, transmembrane.

**
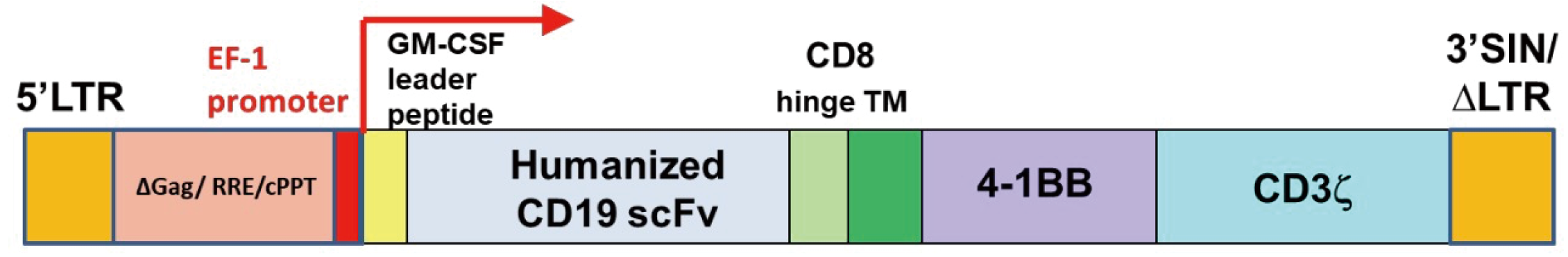
**

**Figure S2. Serum inflammatory factors within 28 days after CART19 infusion.** IL, interleukin; TNF, tumor necrosis factor; IFN, interferon; LDH, lactate dehydrogenase; CRP, C-reaction protein. LD, lymphodepletion.


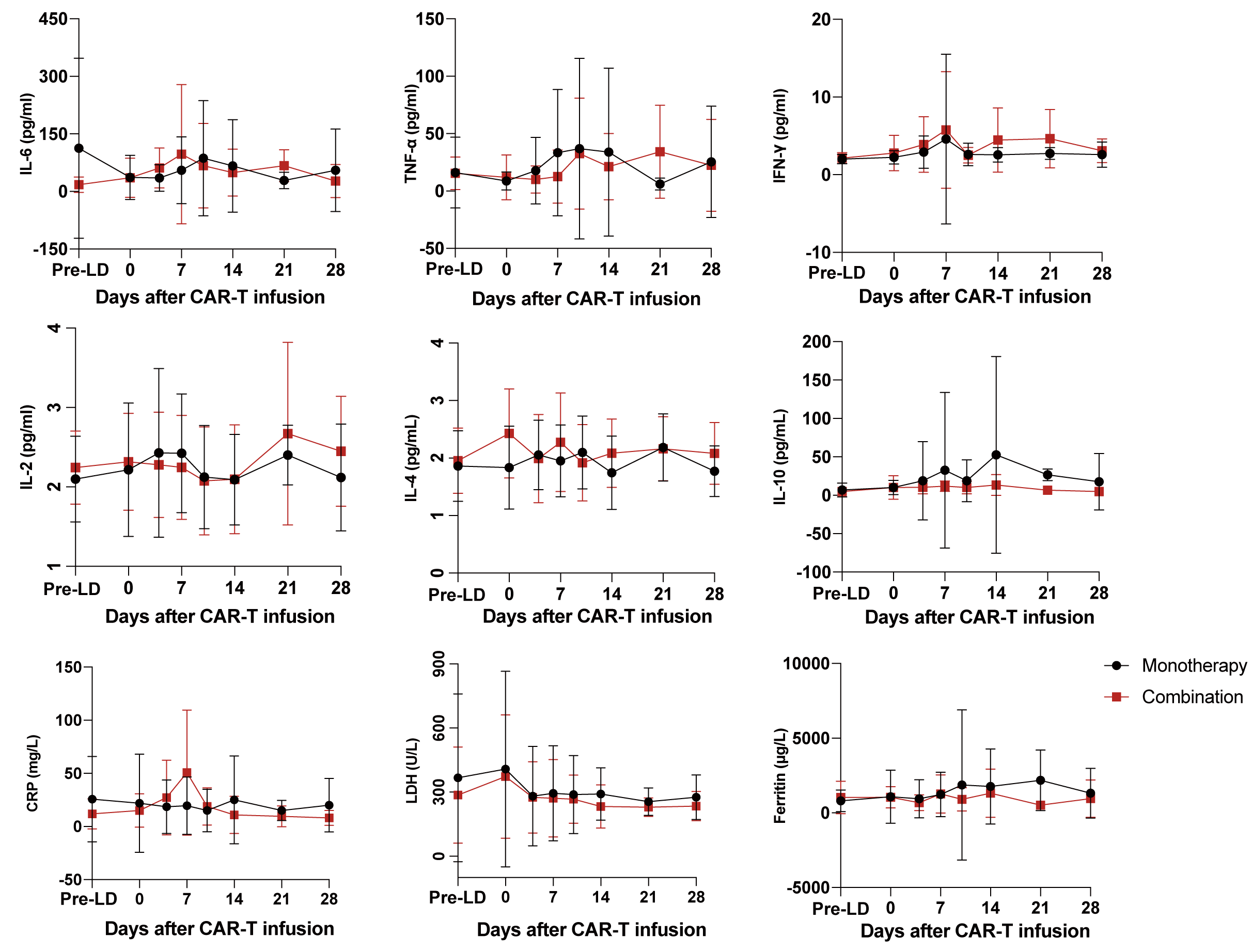


**Figure S3. Hematological recovery of grade 3-4 cytopenias.** (A) The recovery of grade 3-4 cytopenias in all patients. (B) The recovery of grade 3-4 cytopenias between the two groups. Recovery was defined as cytopenias of grade 2 or less. Time to recovery was the time when the criteria were first reached.


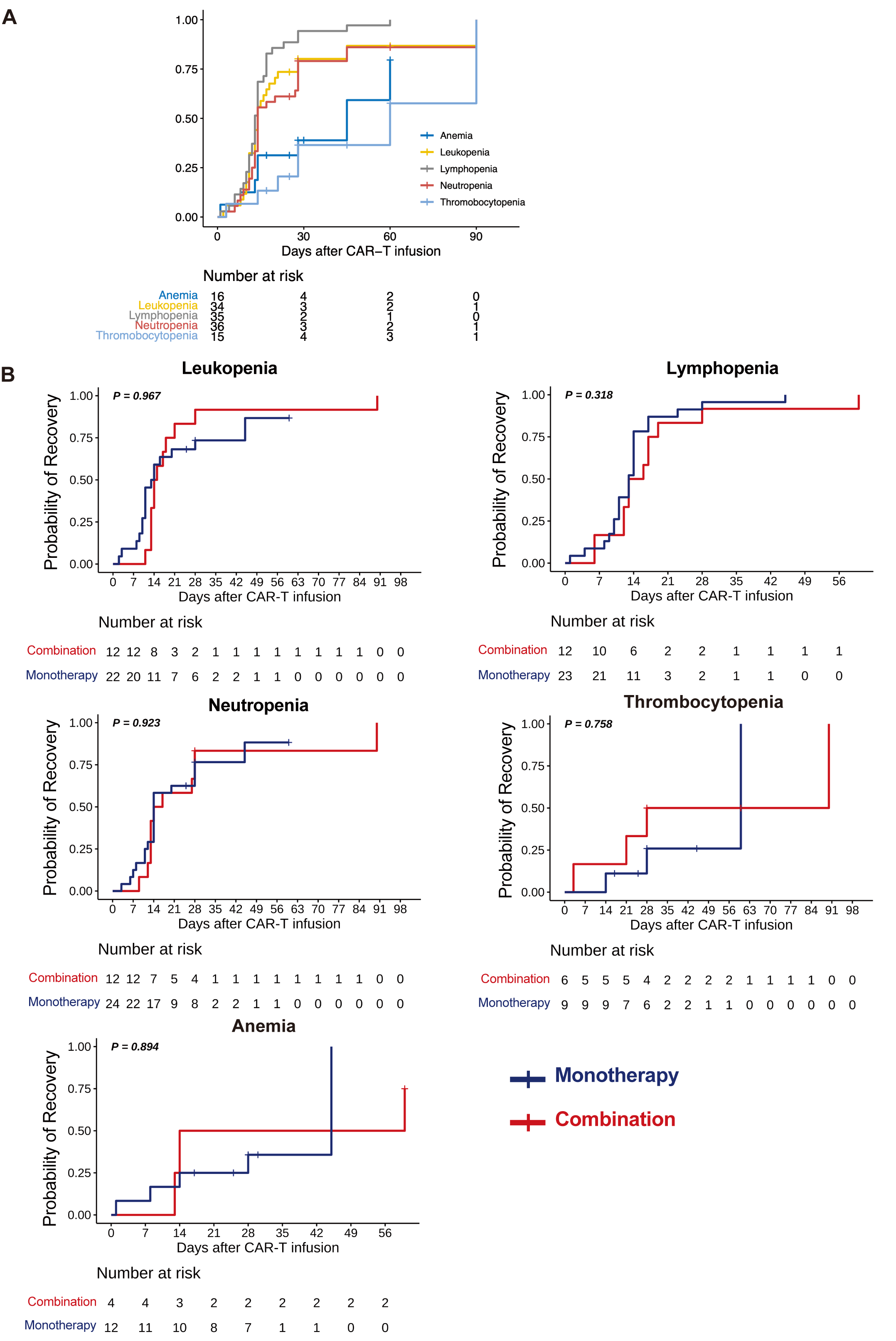


**Figure S4. Changes of immunoglobulin G and B-cell following CART19 infusion.** (A) Patients in the monotherapy group. (B) Patients in the combination group. Dashed lines indicate the lower normal limit of the corresponding factor. Ig, immunoglobulin; PT, patient; LD, lymphodepletion.


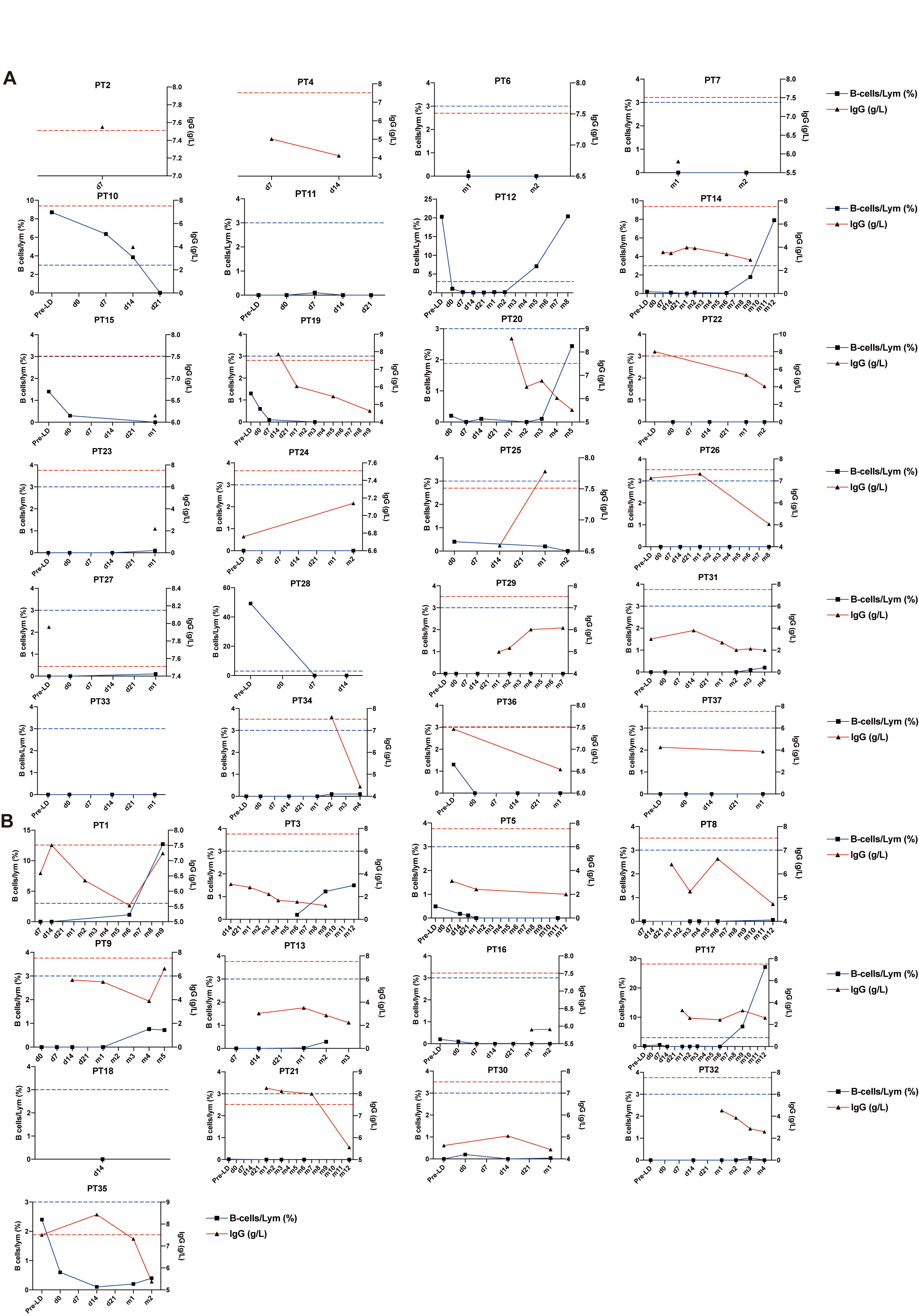


**Figure S5. The effects of three BTKis on OS, PFS, and DOR in the combination group.** OS, overall survival; PFS, progression-free survival; DOR, duration of response.


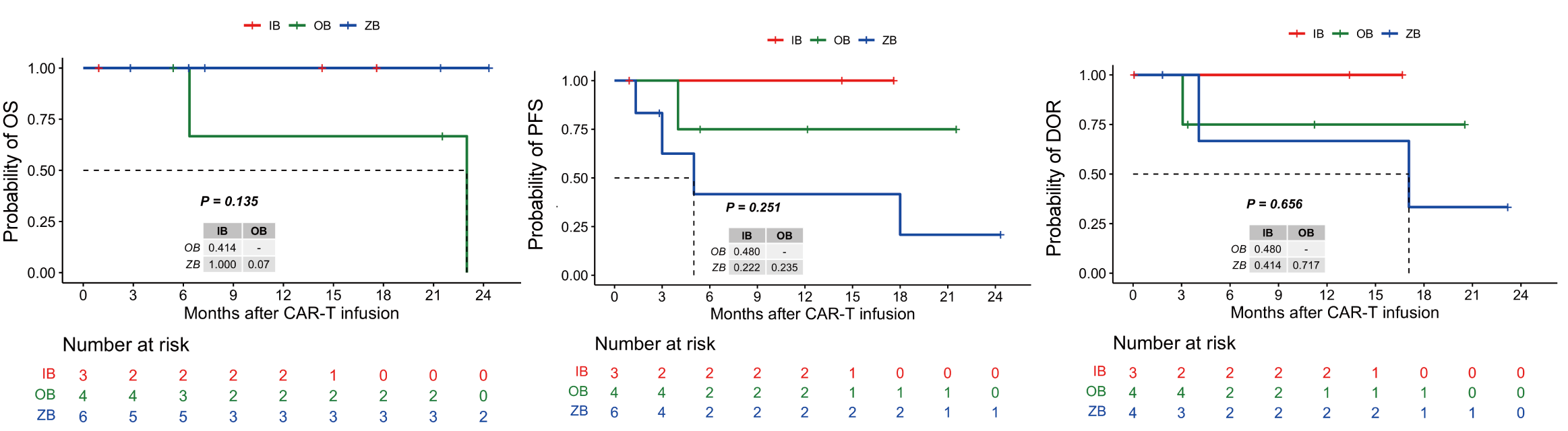


**Figure S6. OS, PFS, and DOR between patients who started receiving BTKi before and after CART19 infusion in combination group.** OS, overall survival; PFS, progression-free survival; DOR, duration of response.


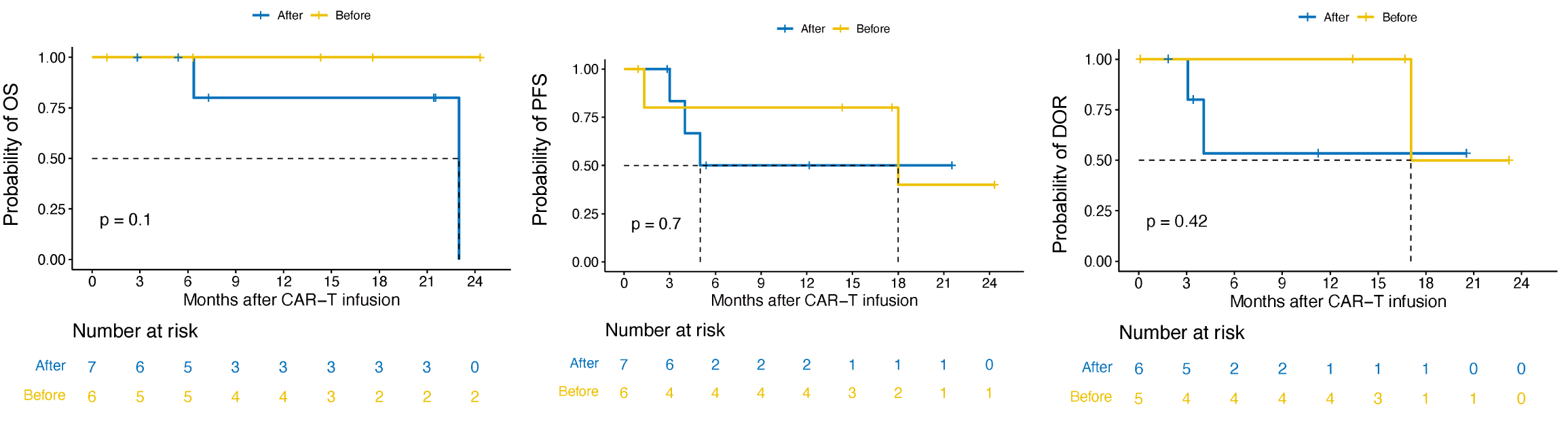


**Figure S7. The median transduction efficiency and the proportion of CD4^+^ or CD8^+^ in CART19 products.**


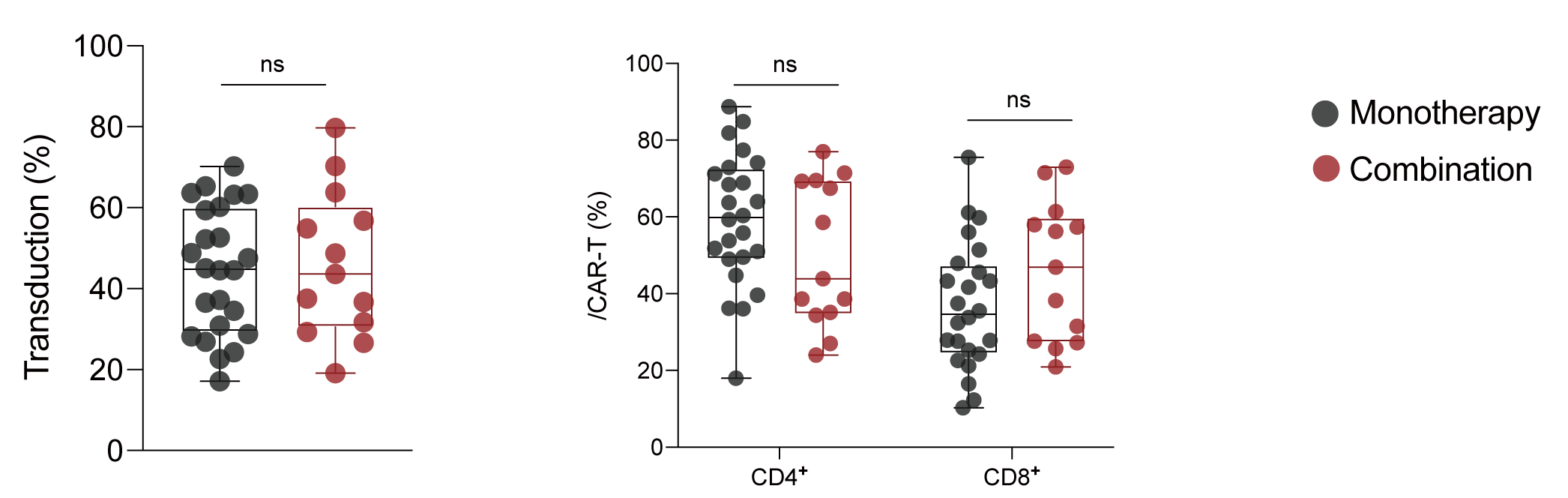


**Figure S8. The peak CART19 and the area under the expansion curve within the first 28 days by the FCM.** FCM, flow cytometry.


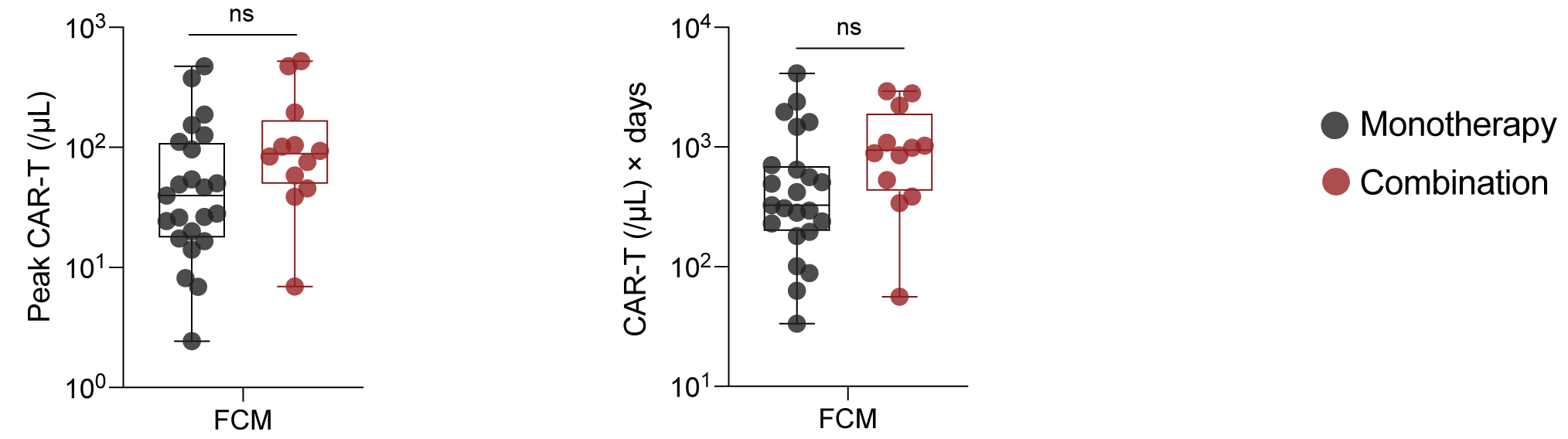


**Figure S9. The T cell differentiation and exhaustion at the two timepoints in the monotherapy group.**  LD, lymphodepletion; m, month.


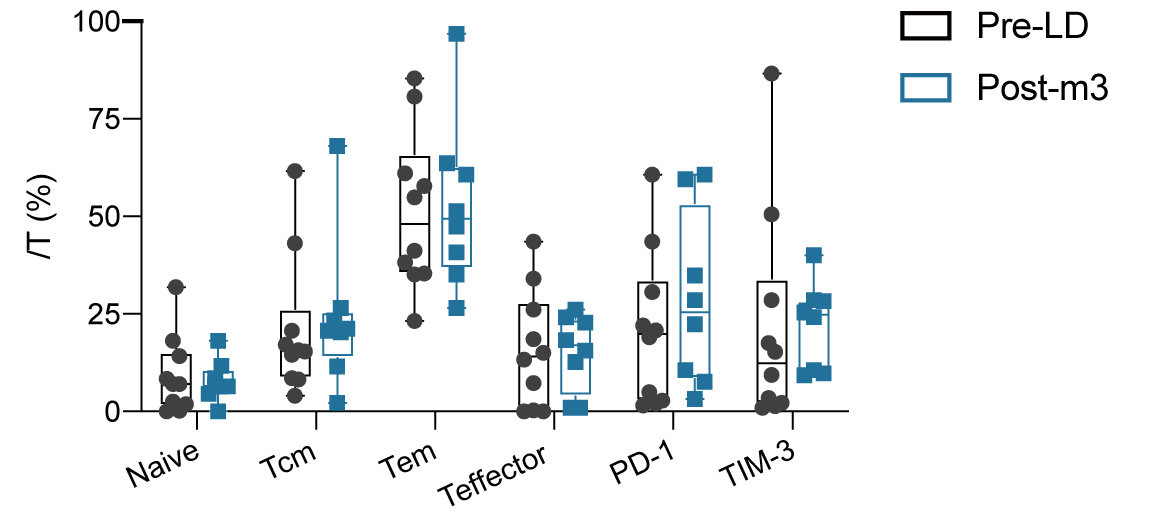


**Figure S10. The expression of marker genes for annotating all cell clusters (A) and T cell clusters (B).**


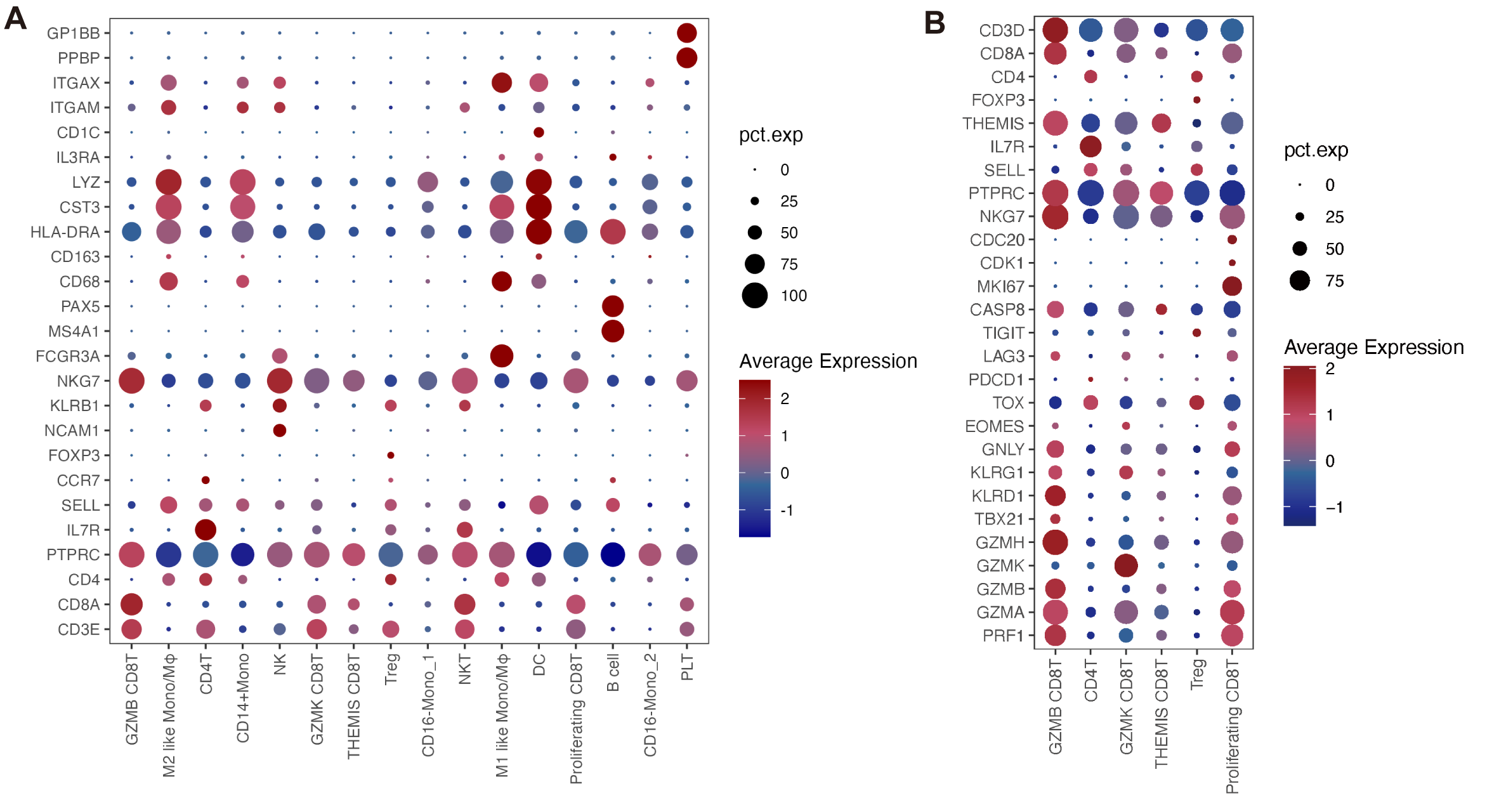


**Figure S11. Volcano plot of differentially expressed genes between cluster 0 and cluster 5.** NS, not significant.


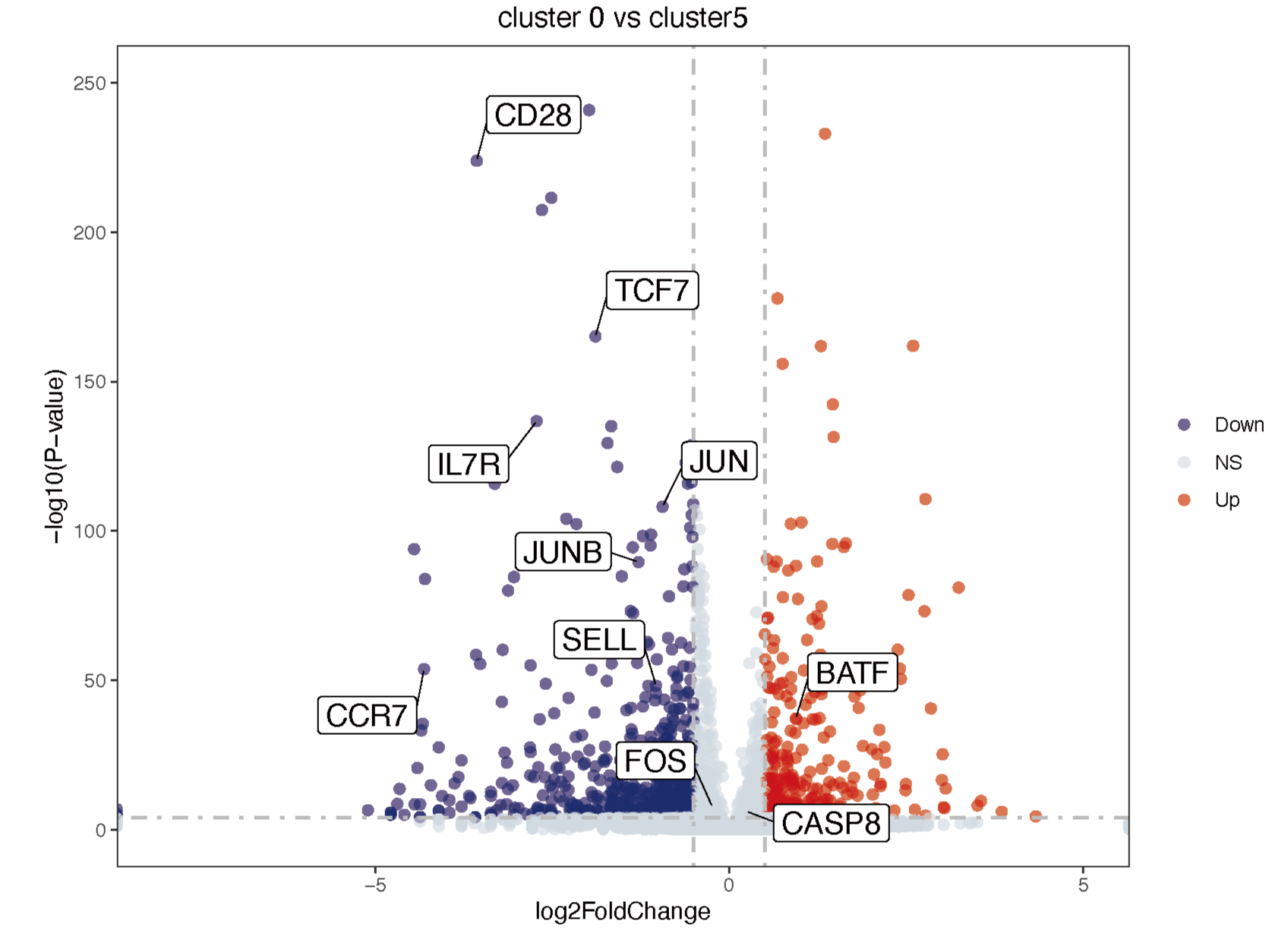


**Figure S12. Upregulated differential expression genes between cluster 6 and the other T cell clusters were enriched based on GO database.** Enriched results within the red box were associated with T cell receptor signaling pathways.


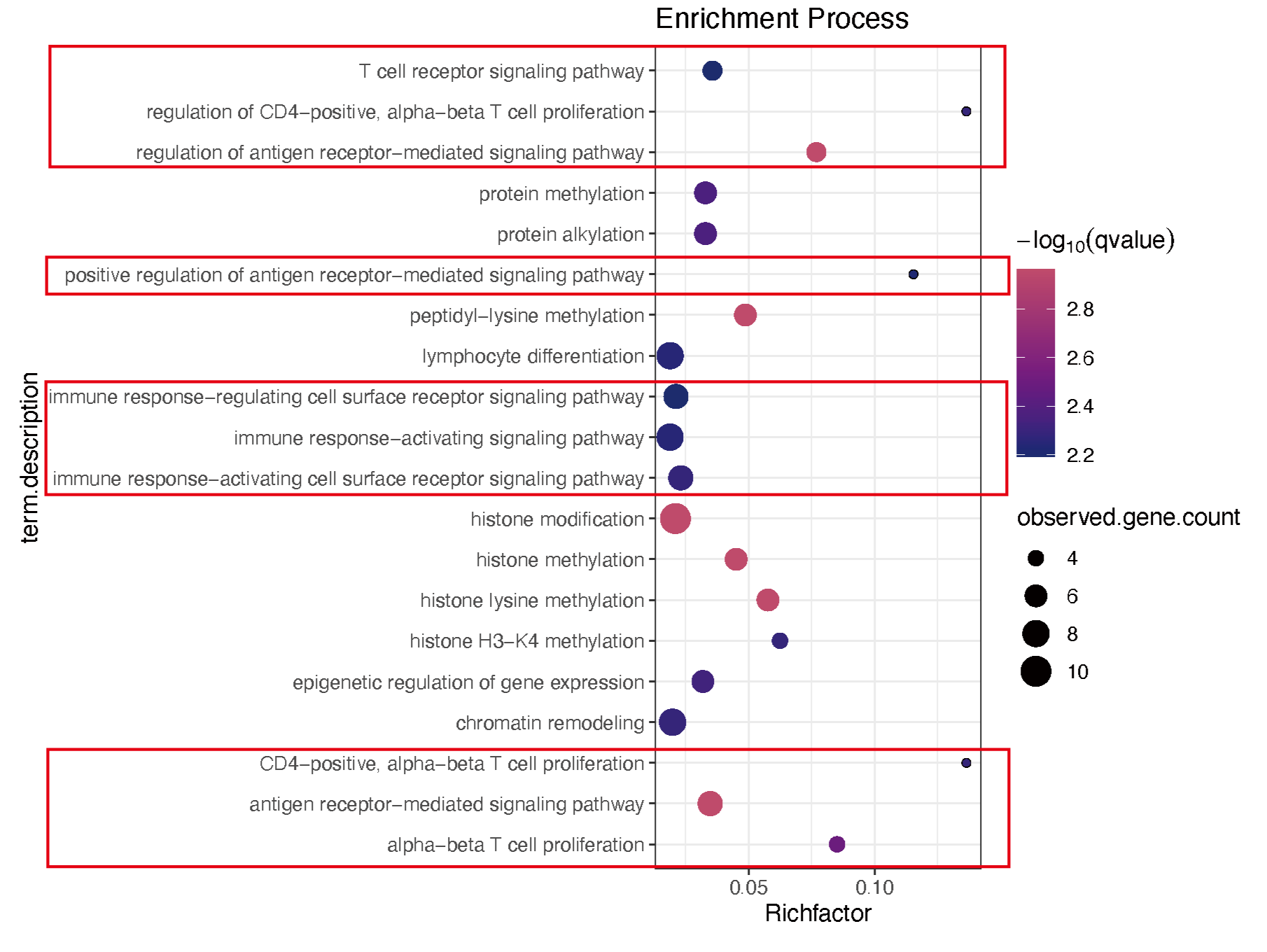

Supplement: Supplementary file 1 — Data S1: cam471321‐sup‐0001‐Supinfo.zip. [file CAM4-14-e71321-s001.zip › cam471321-sup-0001-FigureS1-S12@Supplementary Figures.docx]
