# Supplementary material for: BTK Inhibitor Synergizes With CD19‐Targeted Chimeric Antigen Receptor‐T Cells in Patients With Relapsed or Refractory B‐Cell Lymphoma: An Open‐Label Pragmatic Clinical Trial
Source: Cancer Med. 2025 Oct 22;14(20):e71321. doi: 10.1002/cam4.71321 (PMC12541673; doi:10.1002/cam4.71321)
Supplement: Supplementary file 1 — Data S1: cam471321‐sup‐0001‐Supinfo.zip. [file CAM4-14-e71321-s001.zip › cam471321-sup-0002-Supinfo1@Supplementary Method.docx]

**Supplementary method
Manufacture Standard for CAR-T cell**

1. Appearance and microscopy: The liquid is transparent, no mass, no impurities.
2. Viability: ≥ 70%.
3. T cell purity: ≥ 90%.
4. Transduction rate: ≥ 10%.
5. Microbiological control: negative.
6. Endotoxin: ≤ 0.25 EU/mL.
7. Mycoplasma testing: negative.
