# Supplementary material for: BTK Inhibitor Synergizes With CD19‐Targeted Chimeric Antigen Receptor‐T Cells in Patients With Relapsed or Refractory B‐Cell Lymphoma: An Open‐Label Pragmatic Clinical Trial
Source: Cancer Med. 2025 Oct 22;14(20):e71321. doi: 10.1002/cam4.71321 (PMC12541673; doi:10.1002/cam4.71321)
Supplement: Supplementary file 1 — Data S1: cam471321‐sup‐0001‐Supinfo.zip. [file CAM4-14-e71321-s001.zip › cam471321-sup-0003-TableS1-S3@Supplementary Tables.docx]

**Supplementary tables S1-S3**

| **Table S1. Detailed information of antibodies used in the study** | | | | |
| --- | --- | --- | --- | --- |
| **Antibodies** | | **Manufacturer** | **Cat Number** | |
| Biotinylated human CD19 | | ACRO Biosystems | CD9-H82E9 | |
| PE-streptavidin | | BioLegend | 405203 | |
| APC anti-human CD3 | | BioLegend | 317318 | |
| FITC anti-human CD4 | | BioLegend | 317408 | |
| PerCP-Cy5.5 anti-human CD8 | | BioLegend | 344708 | |
| FITC anti-human CD45 | | BioLegend | 304006 | |
| PE anti-human CD19 | | BioLegend | 302208 | |
| BD Horizon™ Fixable Viability Stain 440UV | | BD Biosciences | 566332 | |
| PerCP-Cy5.5 anti-human CD3 | | BioLegend | 300328 | |
| FITC anti-human CD4 | | BioLegend | 317408 | |
| PE-Cy7 anti-human CD8 | | BioLegend | 344712 | |
| BV510 anti-human CD62L | | BioLegend | 304844 | |
| BV421 anti-human CD45RA | | BioLegend | 304130 | |
| APC anti-human TIM-3 | | BioLegend | 345012 | |
| BV605 anti-human PD-1 | | BioLegend | 329924 | |
| **Table S2. Primers for detect CAR by droplet digital PCR** | | | |  |
|  | **Sequence** | | |  |
| Forward primer | AGAGGAAGATGGCTGTAG | | |  |
| Reverse primer | CTGCTGAACTTCACTCTC | | |  |
| Probe | FAM-CACATCCTCCTTCTTCTTCTTCTGG-TAMRA | | |  |

**Table S3. Characteristics of treated patients**

| **Patient** | **Disease type** | **Ann Arbor stage** | **BM involvement** | **Dual expressor lymphoma*** | **ECOG** | **Prior therapy lines** | **Prior auto-HSCT** | **Primary refractory** | **Baseline LDH(U/L)** | **BTKi (usage)** |
| --- | --- | --- | --- | --- | --- | --- | --- | --- | --- | --- |
| 1 | DLBCL | IV | No | Yes | 0 | 2 | No | No | 91 | ZB (d-25 to d39; 160mg, bid) |
| 2 | DLBCL | IVE | No | Yes | 1 | 2 | No | Yes | 255 | w/o |
| 3 | DLBCL | IVE | Yes | NA | 1 | 5 | No | Yes | 249 | ZB (d-14 to d133; 160mg, bid) |
| 4 | tFL | IIIE | No | NA | 1 | 3 | No | No | 1890 | w/o |
| 5 | tFL | IV | No | No | 1 | 4 | No | Yes | 595 | OB (d37 to d121; 100mg, qd) |
| 6 | DLBCL | IVE | No | No | 1 | 4 | No | Yes | 224 | w/o |
| 7 | DLBCL | IIE | No | No | 1 | 7 | No | Yes | 198 | w/o |
| 8 | DLBCL | III | No | No | 1 | 3 | Yes | No | 273 | OB (d27 to d89; 100mg, qd) |
| 9 | DLBCL | IIE | No | Yes | 1 | 2 | No | No | 166 | ZB (d3 to d46; 160mg, bid) |
| 10 | FL | IVE | Yes | No | 2 | 9 | No | No | 350 | w/o |
| 11 | DLBCL | IVE | Yes | No | 1 | 4 | No | Yes | 304 | w/o |
| 12 | DLBCL | III | No | No | 0 | 2 | No | Yes | 148 | w/o |
| 13 | DLBCL | IV | No | No | 1 | 5 | No | No | 235 | ZB (d13 to d45; 160mg, qd) |
| 14 | DLBCL | IIIE | No | NA | 2 | 2 | No | Yes | 829 | w/o |
| 15 | DLBCL | IIE | No | NA | 2 | 3 | No | Yes | 584 | w/o |
| 16 | DLBCL | IIIE | No | Yes | 2 | 4 | No | Yes | 817 | OB (d21 to d146; 100mg, qd) |
| 17 | DLBCL | IVE | No | No | 1 | 2 | No | Yes | 120 | IB (d-7 to d83; 280mg, qd) |
| 18 | DLBCL | IIIE | No | No | 1 | 2 | No | Yes | 142 | IB (d-24 to d21; 280mg, qd) |
| 19 | DLBCL | IIIE | No | Yes | 1 | 2 | No | Yes | 226 | w/o |
| 20 | DLBCL | IIE | No | No | 0 | 2 | No | No | 195 | w/o |
| 21 | DLBCL | IV | No | Yes | 1 | 2 | No | Yes | 1437 | IB (d-28 to d15; 280mg, qd) |
| 22 | DLBCL | IV | No | Yes | 1 | 4 | No | Yes | 25 | w/o |
| 23 | tFL | IV | No | No | 2 | 3 | No | Yes | 248 | w/o |
| 24 | DLBCL | IVE | No | No | 1 | 5 | No | No | 181 | w/o |
| 25 | DLBCL | IIIE | No | Yes | 1 | 5 | Yes | Yes | 187 | w/o |
| 26 | FL | IVE | No | No | 0 | 4 | Yes | No | 262 | w/o |
| 27 | DLBCL | IVE | No | No | 1 | 3 | No | Yes | 212 | w/o |
| 28 | MCL | IVE | Yes | No | 2 | 4 | No | Yes | 212 | w/o |
| 29 | FL | IV | No | NA | 1 | 4 | No | No | 195 | w/o |
| 30 | DLBCL | IVE | No | No | 1 | 2 | No | Yes | 215 | ZB (d-62 to d37; 160mg, bid) |
| 31 | DLBCL | IIE | No | Yes | 1 | 2 | No | Yes | 456 | w/o |
| 32 | tFL | IVE | No | No | 2 | 3 | Yes | Yes | 197 | OB (d5 to d135; 100mg, qd) |
| 33 | DLBCL | IVE | No | NA | 1 | 4 | Yes | No | 301 | w/o |
| 34 | DLBCL | IVE | No | Yes | 1 | 2 | No | Yes | 162 | w/o |
| 35 | Burkitt | IIE | No | NA | 0 | 3 | No | Yes | 181 | ZB (d14 to d85; 80mg, bid) |
| 36 | DLBCL | IVE | No | Yes | 0 | 2 | No | Yes | 198 | w/o |
| 37 | FL | IV | Yes | No | 1 | 2 | No | No | 219 | w/o |
| *Lymphoma with overexpression of MYC and BCL2 proteins by immunohistochemistry, regardless of detectable translocation by fluorescence in situ hybridization. d: day after CART19 infusion; ECOG: Eastern Cooperative Oncology Group; BM: bone marrow; HSCT: hematopoietic stem cell transplantation; BTKi: Bruton tyrosine kinase inhibitor; LDH: lactate dehydrogenase; DLBCL: diffuse large B-cell lymphoma; GCB: germinal center B-cell-like; FL: follicular lymphoma; tFL: transformed FL; MCL, mantle cell lymphoma; NA: not available; w/o: without; ZB: zanubrutinib; OB: orelabrutinib; IB: ibrutinib. LDH normally ranges from 120-246 U/L. | | | | | | | | | | |
